# Qualitative analysis of pharmacogenetic applications in pediatric clinical trials registered on ClinicalTrials.gov

**DOI:** 10.3389/fphar.2026.1792180

**Published:** 2026-06-12

**Authors:** Rawan H. Hareeri, Mohammed M. Aldurdunji

**Affiliations:** 1 Department of Pharmacology and Toxicology, Faculty of Pharmacy, King Abdulaziz University, Jeddah, Saudi Arabia; 2 Pharmaceutical Practices Department, College of Pharmacy, Umm Al-Qura University, Makkah, Saudi Arabia

**Keywords:** clinical trials, pediatrics, pharmacogenetics, precision medicine, qualitative analysis

## Abstract

**Background:**

Pharmacogenetics (PGx) may support more individualized pediatric therapy by accounting for genetic variability in drug response, toxicity, and dose requirements. However, the extent and manner in which pharmacogenetic and biomarker-related components are incorporated into pediatric clinical trials remain incompletely characterized.

**Objectives:**

To qualitatively examine how pharmacogenetic and biomarker-related components are represented and operationalized in pediatric clinical trials registered on ClinicalTrials.gov, with emphasis on disease distribution, apparent roles within trial design, and the primary and secondary outcome domains reported in these studies.

**Methods:**

A registry-based qualitative analysis was conducted using ClinicalTrials.gov. Interventional Phase II to IV trials enrolling participants younger than 18 years of age were screened from database inception through July 2025 using pharmacogenetic-related search terms. Eligible studies included pediatric trials incorporating pharmacogenetic or pharmacogenomic components relevant to drug response, efficacy, toxicity, pharmacokinetics/pharmacodynamics, dose optimization, or treatment selection. Included trials were classified by primary clinical disease category, apparent level of pharmacogenetic integration, and primary and secondary outcome domains.

**Results:**

A total of 198 pediatric trials met the inclusion criteria. Infectious diseases and oncology were the most frequently represented primary clinical disease categories, each accounting for 37 (18.7%) of included trials, followed by psychiatry and respiratory diseases at 20 (10.1%) each. Exploratory or observational incorporation of pharmacogenetic or biomarker-related information was the most common pattern, accounting for 121 (61.1%) of trials, whereas guided intervention and decision-informing use accounted for 39 (19.7%) and 25 (12.6%), respectively. At the primary outcome level, clinical efficacy was the dominant domain, accounting for 82 (41.4%) of trials, followed by biomarkers/molecular outcomes at 48 (24.2%). Among secondary outcomes coded as inclusive occurrences, biomarkers/molecular outcomes were the leading category at 116 (27.0%), followed by clinical efficacy at 103 (24.0%).

**Conclusion:**

Pediatric pharmacogenetic trials appear to be advancing but remain largely positioned at an intermediate translational stage. Pharmacogenetic and biomarker-related components were more often exploratory than treatment-guiding, highlighting the need for clearer registry reporting and more explicit trial designs that distinguish exploratory, decision-informing, and clinically actionable PGx roles.

## 1 Introduction

Efforts to improve the safety and efficacy of pediatric pharmacotherapy have increasingly incorporated pharmacogenetic principles. Genetic variation may contribute to interindividual differences in drug disposition, therapeutic response, toxicity, and dose requirements in children, and pharmacogenetic approaches may therefore help refine prescribing and monitoring in pediatric care (Quteineh and Verstuyft, 2010; Barker et al., 2022). At the same time, the broader translation of precision medicine into pediatrics appears to remain incomplete. Although individualized therapeutic approaches are increasingly emphasized, implementation in children has been more limited than in adults and has not progressed uniformly across clinical contexts (Elzagallaai et al., 2023).

A central reason for this limited transferability is that pediatric pharmacogenetics is not simply an extension of adult pharmacogenetics. Across infancy, childhood, and adolescence, developmental changes in drug-metabolizing enzymes, transporters, organ function, and pharmacodynamic systems may alter the clinical expression of genetic variation. As a result, genotype-phenotype relationships identified in adults may not be directly reproducible in children, and the relative importance of ontogeny and genetics may vary across developmental stages (Leeder and Kearns, 2002; Neville et al., 2011). Contemporary work has further emphasized that ontogeny may, in some settings, supersede genetic variation as a determinant of enzymatic function, reinforcing the need for age-specific interpretation rather than direct extrapolation from adult data alone (Allegaert and van den Anker, 2019; Chen et al., 2023).

Despite the growing relevance of pharmacogenetics to pediatric therapeutics, evidence generation in children has remained methodologically heterogeneous and, in many areas, relatively sparse. Progress appears to have been greatest in selected specialties, particularly oncology, where severe disease, established cooperative infrastructures, and comparatively well-characterized drug-gene associations may have facilitated earlier integration. By contrast, pharmacogenetic investigation in other pediatric specialties appears more limited, which may reflect, at least in part, developmental complexity, small and heterogeneous study populations, ethical and operational constraints, and incomplete pediatric-specific validation (Piana et al., 2012; Balevic and Cohen‐Wolkowiez, 2018). Accordingly, the current landscape of pediatric pharmacogenetic research may be expected to be uneven across disease areas rather than uniformly mature, a pattern that appears consistent with broader assessments of pediatric drug development and translational gaps (Neumann et al., 2022; Subasri et al., 2023).

Within this setting, the role of pharmacogenetic information in pediatric trials may also be heterogeneous. In some studies, genetic or biomarker data may be incorporated prospectively to inform dosing, treatment selection, or stratification. In others, such data may be collected in exploratory, correlative, or retrospective ways without a clearly treatment-guiding function. This distinction is important, but it may not always be apparent from registry records alone. Prior work suggests that trial records and related pharmacogenomic documentation often incompletely specify the genes or variants under investigation, the analytic methods used, or the intended clinical role of the biomarker component, thereby limiting the interpretability of apparent pharmacogenetic activity (Sing et al., 2015; Green et al., 2016). Similar concerns have been noted in registry-based analyses of pharmacogenomic trials, where pharmacogenetic content may be identifiable but not always sufficiently described to permit confident classification of its intended use (Nogueiras-Álvarez, 2023).

Clinical trial registries may therefore provide a useful, although inherently limited, means of characterizing how pharmacogenetic concepts are represented in pediatric research. Registry-based analyses may help identify the distribution of trials across disease categories, the apparent ways in which pharmacogenetic approaches are incorporated, and broad patterns in study objectives and reported outcomes. Importantly, they may also help distinguish whether pharmacogenetic and biomarker-related components are positioned as exploratory measures, decision-informing elements, monitoring tools, or treatment-guiding components within trial design. However, registries capture declared study information rather than the full substantive conduct or translational maturity of a trial, and incomplete reporting may constrain interpretation, particularly when pharmacogenetic components are described only in general or exploratory terms (Cai et al., 2023; Gregornik et al., 2020).

Accordingly, the present study aimed to qualitatively examine how pharmacogenetic and biomarker-related components are represented and operationalized in pediatric clinical trials registered on ClinicalTrials.gov. The analysis focused on three interrelated aspects of trial design and reporting: the distribution of studies across disease areas, the apparent roles assigned to pharmacogenetic and biomarker-related components, and the primary and secondary outcome domains reported in the registered studies. By distinguishing exploratory, decision-informing, monitoring-related, and treatment-guiding roles, this registry-level analysis provides insight into the translational positioning of pediatric pharmacogenetics within contemporary clinical trial design.

## 2 Methods

### 2.1 Search strategy

The search process was conducted in a stepwise manner. ClinicalTrials.gov was searched from database inception through July 2025 without applying a calendar-year restriction. Records were first filtered to include interventional studies enrolling participants younger than 18 years of age and classified as Phase II, III, or IV, yielding 27,304 pediatric trials. Pharmacogenetic-focused records were then identified by applying the terms “pharmacogenetics,” “pharmacogenomics,” “genetic marker,” “genotyping,” and “biomarker” within the “Other Terms” or “Intervention” fields. Retrieved records were subsequently screened according to the predefined inclusion and exclusion criteria, resulting in a final set of 198 eligible pediatric trials for qualitative analysis.

### 2.2 Inclusion and exclusion criteria

Studies were included if they enrolled pediatric participants and incorporated a pharmacogenetic/pharmacogenomic (PGx) component. For the purposes of this analysis, pediatric participants were defined as individuals younger than 18 years of age according to the age eligibility criteria listed in ClinicalTrials.gov. Only interventional Phase II to IV trials were considered eligible for inclusion. Studies were excluded if they did not involve pharmacogenetic elements, focused solely on genetic diagnostics without therapeutic relevance, or enrolled exclusively adult populations. In this study, a pharmacogenetic/pharmacogenomic component was defined as any planned assessment of germline genetic variation, including genotyping, sequencing, or specified pharmacogenetic markers, relevant to drug response, efficacy, toxicity, pharmacokinetics/pharmacodynamics, dose optimization, or treatment selection.

### 2.3 Data extraction

For each eligible trial, data were extracted from registry fields including study title, registration number, trial phase, recruitment status, disease condition, interventions, sex eligibility, age eligibility, enrollment, allocation, intervention model, masking, primary purpose, and reported primary and secondary outcome measures. Information related to pharmacogenetic implementation, such as biomarker stratification, dose adjustment, or toxicity monitoring, was also recorded when available. Genes and genetic variants were not extracted as a formal analytic variable because registry reporting of this information was inconsistent and frequently incomplete across the included trials. All extracted variables were obtained from publicly available trial registry entries on ClinicalTrials.gov, and the complete extracted dataset, including trial identifiers, is provided in Supplementary Data S1.

### 2.4 Disease category classification

For descriptive analysis, each included trial was assigned to a single primary clinical disease category. Classification was based primarily on the ClinicalTrials.gov condition field to identify the principal disease or disorder under study. Because registry condition fields may also contain treatment names, pharmacogenetic terms, complications, or other non-disease descriptors, the study title and brief summary were reviewed when clarification of the primary clinical context was required. Trials were then grouped into broad categories reflecting their main disease focus, including infectious diseases, oncology, psychiatry, respiratory diseases, endocrine/metabolic/genetic disorders, hematology, immunology/autoimmune diseases, neurology, transplant medicine, cardiovascular diseases, pain/perioperative conditions, dental/oral conditions, gastrointestinal/hepatic disorders, and other/unclassified conditions. Classification was based on the primary clinical population rather than the biomarker, intervention, or endpoint under investigation.

### 2.5 Apparent pharmacogenetic integration classification

To characterize how pharmacogenetic or biomarker-related components were incorporated within the included trials, registry records were reviewed in full, including the study title, brief summary, intervention description, condition field, and reported primary and secondary outcome measures. Each trial was then assigned to one category reflecting the apparent level of pharmacogenetic integration described in the registry entry. Trials were classified as guided intervention when pharmacogenetic or biomarker information was explicitly used to guide treatment selection, dosing, or other intervention-related decisions. Trials were classified as decision-informing when genetic or biomarker information was used for stratification, enrichment, subgroup identification, or response prediction without clearly determining intervention assignment or dose. Trials were classified as biomarker-informed monitoring when biomarkers were used primarily for monitoring, surveillance, or response tracking rather than treatment selection. Trials were classified as exploratory or observational when pharmacogenetic or biomarker components were assessed mainly for association, correlation, mechanistic exploration, or *post hoc* interpretation without explicit influence on treatment decisions. Trials were classified as having no explicit PGx/biomarker-guided role when a pharmacogenetic or biomarker element was present but no treatment-guiding or decision-informing function could be identified from the registry record. Classification was applied conservatively and was based only on information explicitly stated in the registry entry.

### 2.6 Outcome domain classification

Primary and secondary outcome measures were extracted from the registry records of all included studies and classified into predefined outcome domains. Primary outcomes were classified at the trial level, and each included trial was assigned to one primary outcome domain according to the principal construct measured. Secondary outcomes were coded as non-mutually exclusive outcome-domain occurrences; accordingly, a single trial could contribute to more than one secondary outcome domain.

Outcome domains included clinical efficacy, biomarkers/molecular outcomes, pharmacokinetic or dosing outcomes, safety or adverse events, functional/physiological/growth outcomes, health services/implementation/economic outcomes, survival, quality of life/patient-reported outcomes, neurocognitive/developmental outcomes, and not classifiable/none specified where applicable. When an outcome could plausibly fit more than one domain, it was assigned according to the most immediate construct measured, prioritizing direct pharmacologic or clinical endpoints over broader mechanistic interpretation. Composite outcomes were classified according to their dominant component in the main synthesis.

### 2.7 Coding procedures

All coding was performed by a single investigator using a predefined extraction and classification framework. Following the initial coding process, all entries underwent a second structured review to verify consistency of disease-category assignment, pharmacogenetic integration classification, and outcome-domain coding. This second-pass review was conducted by the same investigator and was intended to improve internal consistency of the final analytic dataset.

### 2.8 Data synthesis

Descriptive statistics were used to summarize the characteristics of the included trials and the distribution of studies across disease categories. Frequencies and percentages were calculated for study characteristics, primary clinical disease categories, and apparent levels of pharmacogenetic integration. For outcome analyses, frequencies and percentages were calculated separately for primary outcome domains at the trial level and for secondary outcome-domain occurrences, which were non-mutually exclusive and therefore exceeded the total number of included trials. The analyses were intended to describe how pharmacogenetic and biomarker components were represented in registered pediatric trials rather than to evaluate trial outcomes or quantify the effectiveness of pharmacogenetic-guided interventions.

## 3 Results

### 3.1 Study characteristics

The design and eligibility characteristics of the included pediatric-enrolling pharmacogenetic trials are summarized in Table 1. Most trials permitted enrollment of participants of all sexes, with n = 174 (87.9%), whereas female-only and male-only eligibility were uncommon. With respect to age eligibility, trials open to children, adults, and older adults formed the largest subgroup, with n = 91 (46.0%), followed by child-only trials with n = 56 (28.3%) and trials enrolling children and adults without older adults with n = 51 (25.8%). Enrollment size varied, although most trials enrolled fewer than 250 participants. Randomized allocation was reported in n = 100 (50.5%) trials, and parallel assignment was the most frequently reported intervention model, with n = 97 (49.0%). Most trials were conducted without masking, with n = 129 (65.2%) reporting no masking.

**TABLE 1:** Design and eligibility characteristics of included trials (N = 198).

Characteristic	Category	n (%)
Sex eligibility	All sexes	174 (87.9)
​	Female only	15 (7.6)
​	Male only	9 (4.5)
Age eligibility pattern	All ages	91 (46.0)
​	Child only	56 (28.3)
​	Child and adult only	51 (25.8)
Enrollment size	Not reported	8 (4.0)
​	<50	73 (36.9)
​	50–99	40 (20.2)
​	100–249	40 (20.2)
​	250–499	15 (7.6)
​	≥500	22 (11.1)
Trial phase classification*	Phase 2	102 (48.8%)
​	Phase 3	48 (23.0%)
​	Phase 4	59 (28.2%)
Allocation	Randomized	100 (50.5)
​	Non-randomized	26 (13.1)
​	Not applicable	65 (32.8)
​	Not reported	7 (3.5)
Intervention model	Parallel assignment	97 (49.0)
​	Single-group assignment	76 (38.4)
​	Crossover assignment	11 (5.6)
​	Sequential assignment	5 (2.5)
​	Factorial assignment	3 (1.5)
​	Not reported	6 (3.0)
Masking	None (open-label)	129 (65.2)
​	Single	9 (4.5)
​	Double	21 (10.6)
​	Triple	9 (4.5)
​	Quadruple	23 (11.6)
​	Not reported	7 (3.5)

* Phase categories were coded inclusively. Trials with combined phase designations could contribute to more than one displayed phase category. Accordingly, percentages were calculated using 209 displayed phase-classification occurrences as the denominator.

### 3.2 Distribution of trials by primary clinical disease category

The distribution of included trials across primary clinical disease categories is summarized in Table 2. Infectious diseases and oncology were the most frequently represented categories, with n = 37 (18.7%) each. Psychiatry and respiratory diseases followed, with n = 20 (10.1%) each. Endocrine/metabolic/genetic disorders and hematologic conditions each accounted for n = 11 (5.6%), while immunology/autoimmune diseases, neurology, transplant medicine, and other/unclassified conditions each accounted for n = 10 (5.1%). Lower frequencies were observed for cardiovascular disease, pain/perioperative conditions, dental/oral conditions, and gastrointestinal/hepatic disorders.

**TABLE 2:** Distribution of included pediatric pharmacogenetic trials by primary clinical disease category (N = 198).

Disease category	Count	Percent
Infectious diseases	37	18.7%
Oncology	37	18.7%
Psychiatry	20	10.1%
Respiratory	20	10.1%
Endocrine/Metabolic/Genetic	11	5.6%
Hematology	11	5.6%
Immunology/Autoimmune	10	5.1%
Neurology	10	5.1%
Transplant	10	5.1%
Cardiovascular	7	3.5%
Pain/Perioperative	6	3.0%
Dental/Oral	5	2.5%
Gastrointestinal/Hepatic	4	2.0%
Other/Unclassified	10	5.1%

### 3.3 Apparent roles of pharmacogenetic and biomarker components across included trials

The apparent roles of pharmacogenetic and biomarker components across the included trials are presented in Table 3. Exploratory or observational use was the most frequently identified category, accounting for n = 121 (61.1%) trials. Guided intervention was identified in n = 39 (19.7%) trials, whereas n = 25 (12.6%) were classified as decision-informing. Smaller numbers of trials were categorized as having no explicit pharmacogenetic or biomarker-guided role (n = 9, 4.5%) or biomarker-informed monitoring (n = 4, 2.0%).

**TABLE 3:** Apparent operational role of pharmacogenetic and biomarker-related components among included trials (N = 198).

Apparent level of pharmacogenetic integration	Count	Percent
Exploratory or observational	121	61.1%
Guided intervention*	39	19.7%
Decision-informing	25	12.6%
No explicit pgx/biomarker-guided role	9	4.5%
Biomarker-informed monitoring	4	2.0%

* Includes trials in which pharmacogenetic or biomarker information was used to guide treatment selection, dosing, or other intervention-related decisions.

### 3.4 Distribution of primary and secondary outcome domains

The outcome-domain structure of the included trials is summarized in Table 4. At the primary outcome level, clinical efficacy was the most frequently represented domain, accounting for n = 82 (41.4%) trials, followed by biomarkers or molecular outcomes with n = 48 (24.2%). Pharmacokinetic or dosing outcomes, safety or adverse event outcomes, and functional, physiological, or growth-related outcomes were represented at lower but broadly comparable frequencies. Secondary outcomes showed a broader distribution because they were coded as inclusive occurrences rather than mutually exclusive trial-level categories. Biomarkers or molecular outcomes were the most frequently represented secondary domain, with n = 116 (27.0%) coded occurrences, followed by clinical efficacy with n = 103 (24.0%). Safety or adverse event outcomes and functional, physiological, or growth-related outcomes also appeared frequently as secondary domains.

**TABLE 4:** Distribution of primary and secondary outcome domains among included trials.

*Outcome domain*	*Primary outcomes, n (% of trials)*	*Secondary outcomes, n (% of coded occurrences)*
*Clinical efficacy*	82 (41.4)	103 (24.0)
*Biomarkers/molecular*	48 (24.2)	116 (27.0)
*PK/dosing*	21 (10.6)	24 (5.6)
*Safety/adverse events*	20 (10.1)	68 (15.9)
*Functional/physiological/growth*	19 (9.6)	56 (13.1)
*Health services/implementation/economic*	2 (1.0)	16 (3.7)
*Survival*	1 (0.5)	11 (2.6)
*Quality of life/PROs*	—	18 (4.2)
*Neurocognitive/developmental*	—	1 (0.2)
*Not classifiable/none specified*	5 (2.5)	16 (3.7)
*Total*	198 (100.0)	429 (100.0)

Primary outcomes were classified at the trial level, and each included trial was assigned to one primary outcome domain. Secondary outcomes were coded as non-mutually exclusive outcome-domain occurrences; therefore, a single trial could contribute to more than one secondary outcome domain, and the secondary outcome total exceeds the number of included trials.

## 4 Discussion

### 4.1 Overview of the pediatric pharmacogenetic trial landscape

The present study provides a registry-level characterization of how pharmacogenetic and biomarker-related components are represented within pediatric clinical trials registered on ClinicalTrials.gov. Overall, the findings suggest a pediatric pharmacogenetic trial landscape that is active but unevenly distributed across disease areas, with infectious diseases and oncology emerging as the most represented categories at 37 trials each (18.7%). A central finding was that pharmacogenetic information was more commonly positioned as an exploratory or observational component than as a direct determinant of treatment selection, dosing, or trial conduct. This was reflected by the predominance of exploratory or observational integration, identified in 121 trials (61.1%), and by the outcome structure, in which clinical efficacy remained the leading primary outcome domain while biomarker and molecular measures were more prominent among secondary outcomes. These patterns suggest that pediatric pharmacogenetic research has expanded across multiple therapeutic areas but often remains at an intermediate translational stage, in which PGx and biomarker-related information is increasingly incorporated into interventional trial architecture but less frequently used as a prospective determinant of treatment selection, dosing, or trial conduct.

### 4.2 Therapeutic-area distribution and registry-level trial visibility

Trial activity remained concentrated in selected therapeutic areas rather than distributed evenly across pediatric medicine. In the present cohort, infectious diseases and oncology were the most represented categories, followed by psychiatry and respiratory diseases. This pattern is broadly consistent with prior pediatric registry evidence, in which 137 pediatric pharmacogenomic trials were identified and 69% were concentrated within oncology, psychiatry, analgesic, immunology, and infectious disease categories (Kim et al., 2015; Lavertu et al., 2018). The recurrence of oncology, psychiatry, and infectious diseases across the present analysis and prior pediatric registry evidence suggests that pediatric pharmacogenetic research has remained more visible in a relatively stable set of specialties. However, the present distribution also appears less exclusively oncology-centered than broader adult-oriented pharmacogenomic trial landscapes, in which oncology has often been more dominant and infectious diseases less visible (Nogueiras-Álvarez, 2023). This difference may reflect pediatric-specific therapeutic priorities and the limited transferability of some adult-derived precision-medicine frameworks into child-specific trial contexts.

Beyond the more visible categories, most remaining disease areas were only modestly represented in the present registry cohort. This unevenness is not unique to the present analysis, as prior pediatric pharmacogenomic and precision-health literature has also suggested that actionable pharmacogenomic evidence is not equally developed across childhood disease domains (Subasri et al., 2023; Lavertu et al., 2018). Several factors may contribute to this pattern. Disease areas with established research networks, standardized endpoints, available assays, and more mature gene-to-decision pathways may be better positioned to support interventional pharmacogenetic studies. Conversely, areas with less developed pediatric evidence, fewer validated biomarkers, or more heterogeneous clinical endpoints may remain less visible in registries despite plausible pharmacogenetic relevance. This interpretation is consistent with pediatric OMICs literature suggesting that these approaches are most likely to contribute where clinically actionable pathways and pediatric-specific evidentiary frameworks are sufficiently developed (Neumann et al., 2022). Accordingly, lower frequency in the present classification should be interpreted as lower registry-level trial visibility rather than as evidence of limited pharmacogenetic importance.

### 4.3 Predominance of exploratory over treatment-guiding integration

With respect to apparent pharmacogenetic integration, the most notable pattern was the predominance of exploratory or observational incorporation over more direct treatment-guiding use. In the present cohort, exploratory or observational integration accounted for 121 trials (61.1%), whereas guided intervention and decision-informing use accounted for 39 (19.7%) and 25 (12.6%) trials, respectively. This pattern suggests an intermediate translational stage in which pharmacogenetic information is increasingly incorporated into pediatric interventional research but remains more commonly used for association testing, subgroup characterization, or mechanistic interpretation than for prospective treatment selection or dose individualization. Thus, the presence of PGx-related content in trial registration may not necessarily indicate clinically actionable PGx implementation. This interpretation is consistent with biomarker and pharmacogenetic trial-design frameworks in which the evidentiary requirements depend on the intended context of use, and in which predictive or treatment-guiding applications require stronger prospective validation than biomarker measurement alone (Amur et al., 2015; Califf, 2018). It is also supported by regulatory evidence showing that many proposed biomarkers do not progress to qualified decision-grade tools, reflecting persistent challenges related to assay validation, biomarker properties, and context-of-use justification (Bakker et al., 2022). In a wider ClinicalTrials.gov pharmacogenomics analysis, only 44.26% of registrations specified the genes or variants under study, and many entries described pharmacogenetic testing only in broad or incomplete terms (Nogueiras-Álvarez, 2023). Pediatric OMICs literature has likewise characterized these approaches as frequently hypothesis-generating within drug development rather than consistently implementation-directed (Neumann et al., 2022). A similar view is reflected in industry-oriented analyses, which suggest that pharmacogenomic DNA collection is common in clinical development, while the downstream clinical utility of these data often remains uncertain, particularly when exploratory findings cannot be readily confirmed across heterogeneous trials (Bienfait et al., 2021). Conceptual trial-design literature also distinguishes exploratory, enrichment, and stratification roles for biomarkers, indicating that the mere presence of pharmacogenetic content does not in itself imply treatment-guiding implementation (Cheek et al., 2015; Tarantino et al., 2019).

### 4.4 Outcome structure and translational positioning of pharmacogenetic endpoints

The outcome-domain structure points in the same general direction. Clinical efficacy was the leading primary outcome domain, accounting for 82 trials (41.4%), whereas biomarkers and molecular outcomes accounted for 48 (24.2%). Among secondary outcomes coded as inclusive occurrences, biomarkers and molecular outcomes became the most frequent category at 116 occurrences (27.0%), followed by clinical efficacy at 103 (24.0%). Although the literature does not appear to provide a directly comparable quantitative breakdown of this primary-versus-secondary pattern, a similar hierarchy has been described in regulatory and trial-design literature. Piana et al. noted that pharmacogenetic studies were often not designed to assess pharmacogenetics as a primary endpoint and argued that earlier-stage protocols should remain exploratory, whereas later-stage studies should link pharmacogenetic differences to clinical outcomes (Piana et al., 2012). Moaddeb and Haga likewise observed that only a minority of pharmacogenetic randomized trials appeared to use a primary pharmacogenetic endpoint, while many others positioned pharmacogenetic measures as secondary or exploratory components (Moaddeb and Haga, 2013). Regulatory biomarker-qualification evidence also supports this hierarchy: in a review of European Medicines Agency biomarker qualification procedures, biomarkers were proposed for several endpoint roles, including surrogate, co-primary, and key secondary uses, but only a minority progressed to qualification, reflecting the evidentiary burden required before biomarkers can function as decision-grade endpoints (Bakker et al., 2022). In pediatric oncology, pharmacogenomic work has also been described, in some cases, as a secondary aim within cooperative-group trials (Ostrenga et al., 2023). A related perspective is offered by broader pediatric precision-health appraisal frameworks, in which efficacy and safety evidence remain central to judging translational readiness, whereas implementation, economic, and other downstream evidence domains tend to emerge later in the development pathway (Subasri et al., 2023). The present pattern is consistent with this structure, in which conventional clinical endpoints continue to anchor formal trial evaluation, while biomarker and molecular measures are more often used to support mechanistic interpretation, stratified analysis, or later translational development. The shift from biomarkers as the second most frequent primary domain to the most frequent secondary domain may therefore suggest that biomarker information is being incorporated into pediatric trial architecture, but more often as explanatory or hypothesis-refining support than as the principal criterion by which trial success is judged.

### 4.5 Registry reporting and interpretation of pharmacogenetic terminology

The interpretation of pharmacogenetic integration was also influenced by how pharmacogenetic and biomarker-related terminology was reported in registry records. In several trials, pharmacogenetic language was used broadly, while the specific gene, variant, biomarker panel, analytical approach, testing timing, or intended role in trial conduct was not described with sufficient operational detail. This pattern is consistent with prior ClinicalTrials.gov-based evidence showing that pharmacogenomic trial records may identify the presence of PGx activity without consistently specifying the genes or variants under study (Nogueiras-Álvarez, 2023). It also reflects a broader reporting issue, since interpretation of PGx testing depends on transparent specification of the variants or haplotypes assessed, the limitations of the test, and the intended use of the result (Kalman et al., 2016). Accordingly, the presence of PGx-related terminology in a registry entry should not be assumed to indicate a clearly defined gene-drug strategy, decision-informing framework, or treatment-guiding design. This gap in registry-based protocol reporting restricts the extent to which PGx components can be linked to specific drug-response questions or assessed for clinical readiness.

### 4.6 Pediatric-specific constraints on pharmacogenetic translation

The predominance of exploratory incorporation may be partly explained by the developmental and operational context of pediatric pharmacology. Guided intervention and decision-informing roles together accounted for n = 64 (32.3%), leaving explicitly action-oriented pharmacogenetic use in the minority. This is also reflected in the outcome structure, where biomarker and molecular outcomes were more prominent as secondary outcomes than as primary endpoints, suggesting that biomarker evidence often supports mechanistic interpretation rather than serving as the main criterion for trial success. This pattern is consistent with the fact that genotype-phenotype relationships in children may be modified by ontogeny, age-dependent pharmacokinetics, pharmacodynamics, and changing disease biology, thereby limiting direct extrapolation from adult pharmacogenetic findings (Leeder and Kearns, 2002; Chen et al., 2023; Gregornik et al., 2020). Before pharmacogenetic information can be incorporated into treatment-guiding pediatric trial designs, additional age-specific validation may therefore be required. Pediatric trial design is further complicated by small and heterogeneous populations, age-stratified recruitment, sparse-sampling constraints, and the difficulty of defining outcomes that remain clinically meaningful across developmental stages (Balevic and Cohen‐Wolkowiez, 2018; Gregornik et al., 2020). In pediatric oncology, similar challenges have been described, including small populations, variation in outcome measurement, uncertainty regarding the clinical relevance of genetic testing, and caution around pre-emptive dose alteration when preservation of treatment benefit remains central (Bernsen et al., 2020). These factors may help explain why exploratory and correlative designs continue to occupy a prominent place in pediatric pharmacogenetic research.

### 4.7 Methodological limitations of the registry-based analysis

Several limitations should be considered when interpreting these findings. The analysis was restricted to ClinicalTrials.gov and relied on predefined pharmacogenetic-related search terms and searchable registry fields; therefore, relevant studies may have been missed if pharmacogenetic or biomarker-related content was described using alternative terminology, incompletely specified in the registry record, or recorded outside the searched fields. In addition, the analysis was based on registered trial descriptions rather than verified trial conduct, full protocols, or subsequent result reporting, meaning that the apparent level of pharmacogenetic integration reflects how PGx components were described at registration level rather than how they were necessarily implemented in practice. Registry reporting depth also varied across included studies, and the specific gene, variant, biomarker panel, analytical approach, testing timing, or operational role of the pharmacogenetic component was not always described with sufficient detail. This limited reliable gene-level or gene-drug-level synthesis and may have affected classification of the intended PGx role. Disease-category assignment and integration-level coding required investigator judgment, although a structured second review was undertaken to improve consistency. Primary outcomes were coded at the trial level, whereas secondary outcomes were coded as inclusive occurrences; therefore, these descriptive structures are informative but not directly interchangeable. Finally, the study was descriptive and registry-based and was not designed to evaluate treatment effects, clinical validity, clinical utility, or actual implementation outcomes.

## 5 Conclusion

Pediatric pharmacogenetic trial activity appears to be advancing, but the present registry-level analysis suggests that this progress remains uneven in disease representation, outcome positioning, and level of PGx integration. The predominance of exploratory or observational PGx use indicates that many trials remain at an intermediate translational stage, between biomarker data generation and treatment-guiding implementation. By distinguishing exploratory, decision-informing, monitoring-related, and guided-intervention roles, this study provides a registry-level framework for assessing translational maturity in pediatric pharmacogenetics. Future pediatric PGx trials should define the intended role of pharmacogenetic testing more explicitly, including whether it is used for discovery, stratification, dose individualization, safety monitoring, or treatment selection. More complete registry reporting of genes, variants, biomarker panels, testing timing, analytical approaches, and decision points is also needed to support clearer assessment of clinical readiness. Strengthening these elements may help move pediatric pharmacogenetic research toward more actionable, child-specific therapeutic evidence.

## Data Availability

The raw data supporting the conclusions of this article will be made available by the authors, without undue reservation.

## References

[B1] Allegaert K. van den Anker J. (2019). Ontogeny of phase I metabolism of drugs. J. Clin. Pharmacol. 59 (S1), S33-S41. 10.1002/jcph.1483 31502685

[B2] Amur S. LaVange L. M. Zineh I. Buckman‐Garner S. Woodcock J. (2015). Biomarker qualification: toward a multiple stakeholder framework for biomarker development, regulatory acceptance, and utilization. Clin. Pharmacol. Ther. 98 (1), 34–46. 10.1002/cpt.136 25868461

[B3] Bakker E. Hendrikse N. M. Ehmann F. Meer D. S. van der Garcia J. L. Vetter T. (2022). Biomarker qualification at the european medicines agency: a review of biomarker qualification procedures from 2008 to 2020. Clin. Pharmacol. Ther. 112 (1), 69–80. 10.1002/cpt.2554 35137949 PMC9313861

[B4] Balevic S. J. Cohen‐Wolkowiez M. (2018). Innovative study designs optimizing clinical pharmacology research in infants and children. J. Clin. Pharmacol. 58 (S10), S58-S72. 10.1002/jcph.1053 30248192 PMC6310922

[B5] Barker C. Groeneweg G. Zee A. H. M. der Rieder M. Hawcutt D. B. Hubbard T. (2022). Pharmacogenomic testing in paediatrics: clinical implementation strategies. Br. J. Clin. Pharmacol. 88 (10), 4297–4310. 10.1111/bcp.15181 34907575 PMC9544158

[B6] Bernsen E. C. Hagleitner M. M. Kouwenberg T. W. Hanff L. M. (2020). Pharmacogenomics as a tool to limit acute and long-term adverse effects of chemotherapeutics: an update in pediatric oncology. Front. Pharmacol. 11, 1184. 10.3389/fphar.2020.01184 32848787 PMC7421781

[B7] Bienfait K. Chhibber A. Marshall J. Armstrong M. Cox C. Shaw P. (2021). Current challenges and opportunities for pharmacogenomics: perspective of the industry pharmacogenomics working group (I-Pwg). Hum. Genet. 141 (6), 1165–1173. 10.1007/s00439-021-02282-3 34081195 PMC9177658

[B8] Cai L. Patel A. A. Thirunavu V. Hug N. F. Song S. Li J. S. (2023). Characterizing clinical trials in plastic and reconstructive surgery. Ann. Plast. Surg. 90 (5S), S287–S294. 10.1097/sap.0000000000003227 37227408

[B9] Califf R. M. (2018). Biomarker definitions and their applications. Exp. Biol. Med. 243 (3), 213–221. 10.1177/1535370217750088 29405771 PMC5813875

[B10] Cheek D. J. Bashore L. Brazeau D. (2015). Pharmacogenomics and implications for nursing practice. J. Nurs. Scholarsh. 47 (6), 496–504. 10.1111/jnu.12168 26470020

[B11] Chen F. Tuzun F. Sherwin C. M. T. (2023). Editorial: Genetics and ontogeny in precision therapeutics for children. Front. Genet. 14, 1308169. 10.3389/fgene.2023.1308169 37964778 PMC10641871

[B12] Elzagallaai A. A. Barker C. Lewis T. Cohn R. D. Rieder M. (2023). Advancing precision medicine in paediatrics: past, present and future. Camb. Prism. Precis. Med. 1, e11. 10.1017/pcm.2022.14 38550924 PMC10953768

[B13] Green D. Mummaneni P. Kim I. Oh J. Pacanowski M. Burckart G. (2016). Pharmacogenomic information in FDA‐approved drug labels: application to pediatric patients. Clin. Pharmacol. Ther. 99 (6), 622–632. 10.1002/cpt.330 26693845

[B14] Gregornik D. Salyakina D. Brown M. Roiko S. A. Ramos K. S. (2020). Pediatric pharmacogenomics: challenges and opportunities: on behalf of the sanford children’s genomic medicine consortium. Pharmacogenomics J. 21 (1), 8–19. 10.1038/s41397-020-00181-w 32843689 PMC7840501

[B15] Kalman L. Agúndez J. Appell M. L. Black J. Bell G. Boukouvala S. (2016). Pharmacogenetic allele nomenclature: international workgroup recommendations for test result reporting. Clin. Pharmacol. Ther. 99 (2), 172–185. 10.1002/cpt.280 26479518 PMC4724253

[B16] Kim T. Han N. Sohn M. Oh J. M. Lee E. Ji E. (2015). Pharmacogenomic biomarker information in FDA-Approved paediatric drug labels. Basic Clin. Pharmacol. Toxicol. 116 (5), 438–444. 10.1111/bcpt.12341 25348905

[B17] Lavertu A. McInnes G. Daneshjou R. Whirl‐Carrillo M. Klein T. E. Altman R. B. (2018). Pharmacogenomics and big genomic data: from lab to clinic and back again. Hum. Mol. Genet. 27 (R1), R72–R78. 10.1093/hmg/ddy116 29635477 PMC5946941

[B18] Leeder J. S. Kearns G. L. (2002). The challenges of delivering pharmacogenomics into clinical pediatrics. Pharmacogenomics J. 2 (3), 141–143. 10.1038/sj.tpj.6500104 12082583

[B19] Moaddeb J. Haga S. B. (2013). Pharmacogenetic testing: current evidence of clinical utility. Ther. Adv. Drug Saf. 4 (4), 155–169. 10.1177/2042098613485595 24020014 PMC3765014

[B20] Neumann E. Schreeck F. Herberg J. Jacqz Aigrain E. Maitland‐van der Zee A. H. Pérez‐Martínez A. (2022). How paediatric drug development and use could benefit from OMICs: a c4c expert group white paper. Br. J. Clin. Pharmacol. 88 (12), 5017–5033. 10.1111/bcp.15216 34997627

[B21] Neville K. A. Becker M. L. Goldman J. L. Kearns G. L. (2011). Developmental pharmacogenomics. Pediatr. Anesth. 21 (3), 255–265. 10.1111/j.1460-9592.2011.03533.x 21320234

[B22] Nogueiras-Álvarez R. (2023). Pharmacogenomics in clinical trials: an overview. Front. Pharmacol. 14, 1247088. 10.3389/fphar.2023.1247088 37927590 PMC10625420

[B23] Ostrenga A. Thackray J. McLearan H. H. Mulieri K. M. Bisaccia E. K. Militano O. (2023). Children’s oncology group’s 2023 blueprint for research: pharmacy. Pediatr. Blood Cancer 70 (S6), e30581. 10.1002/pbc.30581 37460409 PMC10529855

[B24] Piana C. Surh L. Fürst‐Recktenwald S. Iolascon A. Jacqz‐Aigrain E. Jonker I. (2012). Integration of pharmacogenetics and pharmacogenomics in drug development: implications for regulatory and medical decision making in pediatric diseases. J. Clin. Pharmacol. 52 (5), 704–716. 10.1177/0091270011401619 21566202

[B25] Quteineh L. Verstuyft C. (2010). Pharmacogenetics in immunosuppressants: impact on dose requirement of calcineurin inhibitors in renal and liver pediatric transplant recipients. Curr. Opin. Organ Transpl. 15 (5), 601–607. 10.1097/MOT.0b013e32833de1d0 20720493

[B26] Sing C. Cheung C. Wong I. C. K. (2015). Pharmacogenomics – how close/far are we to practising individualized medicine for children? Br. J. Clin. Pharmacol. 79 (3), 419–428. 10.1111/bcp.12338 25855823 PMC4345952

[B27] Subasri M. Cressman C. Arje D. Schreyer L. Cooper E. Patel K. (2023). Translating precision health for pediatrics: a scoping review. Children 10 (5), 897. 10.3390/children10050897 37238445 PMC10217253

[B28] Tarantino P. Trapani D. Morganti S. Ferraro E. Viale G. D’Amico P. (2019). Opportunities and challenges of implementing pharmacogenomics in cancer drug development. Cancer drug Resist (Alhambra, Calif). 2 (1), 43–52. 10.20517/cdr.2018.22 PMC9019172 35582141

